# A Field-based Approach to Support Improved Diabetes Care in Rural States

**Published:** 2005-09-15

**Authors:** Todd S Harwell, Elizabeth A Johnson, Janet M McDowall, Carrie S Oser, Marcene K Butcher, Dorothy Gohdes, Steven D Helgerson, Wanda L Webb, Linda L Chasson, Joseph R Grandpre, Madhavi I Marasinghe, Erin M O’Leary

**Affiliations:** Montana Department of Public Health and Human Services; Montana Diabetes Project, Montana Department of Public Health and Human Services, Helena, Mont; Montana Diabetes Project, Montana Department of Public Health and Human Services, Helena, Mont; Montana Diabetes Project, Montana Department of Public Health and Human Services, Helena, Mont; Montana Diabetes Project, Montana Department of Public Health and Human Services, Helena, Mont; Montana Diabetes Project, Montana Department of Public Health and Human Services, Helena, Mont; Montana Diabetes Project, Montana Department of Public Health and Human Services, Helena, Mont; Wyoming Diabetes Prevention and Control Program, Wyoming Department of Health, Cheyenne, Wyo; Wyoming Diabetes Prevention and Control Program, Wyoming Department of Health, Cheyenne, Wyo; Wyoming Diabetes Prevention and Control Program, Wyoming Department of Health, Cheyenne, Wyo; Montana Diabetes Project, Montana Department of Public Health and Human Services, Helena, Mont; Energy and Environmental Research Center, University of North Dakota, Grand Forks, ND

## Abstract

**Introduction:**

Diabetes care is a challenge in rural areas where primary care practices are faced with limited resources, few clinical information systems, and relative isolation from education programs and diabetes centers with multispecialty teams. This report describes an effective field-based approach to support improved care for patients with diabetes in primary care practices in rural states.

**Methods:**

A collaborative effort between diabetes prevention and control programs in Montana and Wyoming and the University of North Dakota was established to provide support to rural primary care practices for improvement in diabetes care. Field teams from each state diabetes program approached primary care practices. After assessment and orientation of office staff, a computer-based registry was established in each practice. Baseline data were collected in 1997 in Montana and in 1998 in Wyoming; follow-up occurred on July 31, 2004. Health department staff provided ongoing technical support for implementing and evaluating quality-improvement interventions.

**Results:**

Forty primary care practices, providing care to more than 7000 patients with diabetes, participated in this quality-improvement effort at follow-up. Of the 37 primary care practices participating in the quality-improvement program for 6 or more months at follow-up, there were significant improvements in Montana in rates of hemoglobin A1c testing, blood glucose control, low-density lipoprotein cholesterol testing, foot and dilated retinal examinations, and pneumococcal vaccinations, and there were significant improvements in pneumococcal vaccinations in Wyoming.

**Conclusion:**

A field-based approach in which individual practices maintain and use their own registries for both clinical care and quality improvement with ongoing support is a sustainable and an effective strategy for improving diabetes care for rural populations.

## Introduction

Clinical trials have demonstrated that patients with diabetes who achieve optimal blood glucose, blood pressure, and cholesterol levels are much less likely to experience complications than those who do not achieve recommended treatment targets (1-3). The complexity of diabetes care has increased markedly over the past decade as important studies have influenced clinical practice (4). Screening for diabetes-related complications, preventive services, self-management education, and counseling must now be integrated with primary care visits to manage blood glucose, blood pressure, and cholesterol levels. Delivery system design, clinical information systems, and self-management support are critical processes that have been used to improve care and outcomes for patients with diabetes (5,6). Large health care systems often have disease management programs that allow clinicians to track patients and evaluate how well they achieve targets and receive preventive services (6,7). Federal health care agencies such as the Veterans Administration and the Indian Health Service have also organized ways to use clinical data at local levels to coordinate, manage, and improve diabetes care and outcomes for their patient populations (8-10). Federally qualified Community and Migrant Health Centers, funded by the Bureau of Primary Health Care, have used a diabetes registry designed specifically for quality improvement (11).

Diabetes care is a challenge in rural areas where primary care practices are faced with limited resources, few clinical information systems, and relative isolation from diabetes education programs and diabetes centers with multispecialty teams. Technology and support for clinical information systems are often too costly and complicated for rural primary care practices, and ongoing support for quality improvement typically found in managed care organizations is often nonexistent. Although several studies found diabetes care in rural settings to be suboptimal (12-16), other studies demonstrate improvements in both the processes and the intermediate clinical outcomes from diabetes care in rural patient populations (17,18).

Compared with other states, Montana and Wyoming are large geographically and have relatively small populations. In Montana in 2000, the total population of 902,195 lived in an area of 147,042 sq miles with a population density of 6.2 people per sq mile (19). Of the 56 counties in Montana, 48 are defined as *frontier* (fewer than 6 people per sq mile). Similarly, in Wyoming in 2000, the population density was 5.1 people per sq mile with a total population of 493,782 living in a state with 97,814 sq miles. Of the 23 counties in Wyoming, 16 are defined as frontier. Overall, Montana and Wyoming cover 6% of the total landmass of the United States. Based on 2003 prevalence estimates for adults with diabetes in Montana (5.5%) and Wyoming (5.8%), there are approximately 36,967 adults with diagnosed diabetes in Montana and 21,165 adults with diagnosed diabetes in Wyoming (20). 

This report describes the experience and outcomes of a collaboration between two state health departments and the University of North Dakota working with primary care practices in Montana and Wyoming to implement and sustain a successful diabetes quality-improvement effort.

## Methods

The Montana and Wyoming Diabetes Prevention and Control Programs, in collaboration with the Energy and Environmental Research Center at the University of North Dakota, developed a simple computerized registry called the Diabetes Quality Care Monitoring System (DQCMS) to support primary care clinicians in tracking key elements of care for their patient populations with diabetes. These statewide quality-improvement efforts began in 1997 in Montana and in 1998 in Wyoming.

### Establishing and maintaining diabetes registries

The quality-improvement coordinators from both state diabetes programs identified primary care practices potentially interested in participating in this quality-improvement effort. State diabetes program staff actively recruited some practices, and others identified themselves as interested in participating (passive recruitment). Before establishing the registry at each practice, state diabetes program staff conducted an assessment with the practice's clinical and administrative staff to ensure that the practice was prepared to maintain its registry and was interested in implementing interventions to improve care for its patients with diabetes. No funding was provided to primary care practices participating in this program, and there were no direct costs for these practices to participate.

To establish the DQCMS, all patients were identified who had one or more clinic visits in the past year for which a claim included a diabetes diagnosis code (*ICD-9-CM* 250.0–250.9). A small team from the Montana and Wyoming state diabetes programs verified the diagnosis and abstracted demographic and clinical information from the medical records of patients with diabetes who were at that time being followed by the practice. Patients with diabetes who resided in nursing homes were excluded. The most recent date and result of key indicators of diabetes care such as hemoglobin A1c (HbA1c) tests, blood pressure measurements, low-density lipoprotein cholesterol (LDL-C) tests, urinalyses, foot and dilated retinal examinations, and pneumococcal vaccinations were noted and entered into the DQCMS registry. Certain diabetes preventive care indicators were selected for further evaluation to correspond with national *Healthy People 2010* objectives for diabetes (21). Blood glucose control was evaluated by assessing the proportion of patients whose last HbA1c value was less than 8.0%.

The Montana and Wyoming diabetes programs provided support to practices by helping to establish the registry, promoting the implementation of quality-improvement interventions through regular site visits, providing technical assistance in developing diabetes education programs recognized through the American Diabetes Association, and assisting practices to evaluate their quality-improvement efforts. The University of North Dakota programmed and maintains the DQCMS registry. After the baseline registry was established, practice staff added information about newly identified patients with diabetes as they presented for care. The diabetes coordinators at each practice maintained the registries. 

The Figure summarizes the roles of the state diabetes programs and primary care practices in the quality-improvement program.

**Figure:** Key steps in the partnership to establish and maintain diabetes care registries between state diabetes programs and primary care practices, Montana and Wyoming, 2004.

**Table:** 

	**State Diabetes Programs (Nurses and Other Staff):**	**Physician Office (Physicians, Nurses, and Other Staff):**	**Primary Focus:**
** Installation of Registry**	Supply software (no cost to the practice) Identify patients with diabetes (using billing data for initial selection) Abstract data from medical records Enter data into practice-based computer Train practice staff	Supply computer and printer Provide access to billing data and medical records Confirm interest in using computer-based registry	Prepare for quality-improvement activity
**Office Use for Patient Care**	Maintain regular telephone contact with practice staff Conduct regular on-site visits to review and support use of software by practice staff Review *Population Practice Profile *to help identify population-based opportunities to improve care Identify need for and arrange continuing education for clinicians	Review and update patient profile at each patient visit Recognize services due or undesirable metabolic values Take appropriate clinical action	Improve care one patient at a time
**Special Quality Improvement Projects**	Publish *Quality Improvement Report* on a quarterly basis Aggregate and analyze data from quarterly summary report Assist with design and evaluation of quality-improvement projects	Identify and select quality-improvement topic(s) Implement quality-improvement projects Recognize special efforts of practice staff members	Improve care for the population of patients (sometimes for selected subgroups of the population)

### Changes in diabetes care delivery

Primary care practices used preprogrammed reports generated by the DQCMS and technical support from the state quality-improvement coordinators to make a number of changes in the delivery of diabetes care and education. The reports allowed each practice to assess and monitor the health status of its patient population and to identify subgroups of patients in need of services (e.g., patients who used tobacco; patients with elevated HbA1c, blood pressure, or LDL-C levels). Several practices targeted subgroups of patients through mail or telephone outreach; others mailed letters reminding patients about the need for influenza and pneumococcal vaccinations. Several practices used the DQCMS reports to mail educational materials emphasizing the "ABCs" of diabetes (i.e., HbA1c, blood pressure, and LDL-C levels) to each patient being followed in their practice along with personalized health information and current laboratory results.

Additionally, the registry generated one-page patient profiles, which were placed in each patient's medical record to highlight services due at the next office visit and measure patient progress toward achieving clinical practice recommendations. This sheet became a template for updating the registry, thus making current information available for each subsequent visit.

As part of the quality-improvement effort, each participating practice provided a quarterly summary report to each state diabetes program. The aggregate report, the* Population Practice Profile*, provided the total number of patients with diagnosed diabetes in each practice and number and proportion of patients receiving preventive care services and meeting selected clinical outcomes. The state diabetes programs in turn aggregated the data across practices and distributed the* Quality Improvement Report *to each participating practice. The report summarized the level of care across all sites and provided a yearly benchmark for each indicator. Additionally, the *Quality Improvement Report* enabled an individual practice to compare itself against other practices.

### Program evaluation

Data analyses were conducted using SPSS version 11.0 (SPSS Inc, Chicago, Ill). To evaluate the impact of this program, we compared the delivery of selected preventive care services and clinical outcomes for patients in the 37 practices that participated in the quality-improvement program for 6 or more months from baseline through follow-up. Chi-square tests were used to compare changes in the proportion of patients meeting these indicators from the date of initial participation in the quality-improvement program (baseline) through July 31, 2004 (follow-up). Wilcoxon signed rank tests were used to compare changes in the median percentage across each practice at baseline and follow-up. Improvements in median and overall percentage of patients with diabetes receiving preventive care services and meeting clinical outcomes were significant at α = .05.

### Results

At follow-up, 24 primary care practices in Montana and 16 primary care practices in Wyoming participated in this quality-improvement program (Table 1). These practices provided care to 5859 patients with diagnosed diabetes in Montana and 2267 patients with diagnosed diabetes in Wyoming. In Montana, practices participated in the program for a median of 32 months (range, 1–81 months); in Wyoming, practices participated for a median of 38 months (range, 10–70 months). Of the 37 practices participating for 6 or more months, 38% of practices in Montana were group or individual primary care practices, and 94% of practices in Wyoming were group or individual primary care practices.

Of the primary care practices participating in the quality-improvement program for 6 or more months in Montana, there were significant improvements in HbA1c testing, glycemic control (i.e., last HbA1c level was less than 8.0%), LDL-C testing, foot and dilated retinal examinations, and pneumococcal vaccinations (Table 2). In primary care practices participating for 6 or more months in Wyoming, there was a significant improvement in pneumococcal vaccination rates.

### Discussion

Our findings suggest that this unique field-based approach between state health departments and primary care practices in the two rural states has been successful. With the technical assistance and support from the state health department teams, primary care practices have implemented systems changes, organized outreach, and increased educational opportunities for their patients with diabetes. Several groups of diabetes educators providing educational support to primary care practices (two sites in Montana and 12 in Wyoming) have also used DQCMS to track patients, although their data are not included in this report.

We have previously reported improvements in care, outcomes, and reduced self-management barriers in small groups of primary care practices in Montana (17,18,22,23); however, the effort described in this report indicates that improvements in care can be made statewide for a large proportion of patients with diabetes.

### Sustained success

There are a number of factors that we feel are important for the sustained success of this approach. First, the DQCMS was developed to be simple to use and maintain. The number of data fields is limited to the key elements of diabetes care, and there are only two data entry screens, only one of which is used for the day-to-day clinical data. Thus, the burden of data entry for practice staff is kept to a minimum. Second is the onsite support furnished by the state diabetes program teams to the clinical staff to establish the registry and target ongoing quality-improvement efforts. The state diabetes programs provided mentoring support to improve the diabetes education skills of selected clinical staff in ways that allowed them to participate at their own pace and define their own goals. This type of support would not have been available through other sources. Third, the ongoing onsite support for individual quality-improvement projects was also key. Managed-care plans often provide quality-improvement services to primary care practices within their organization, and several have documented their successful efforts to improve diabetes care (8,24). Montana and Wyoming, however, have relatively few managed-care plans, and those that exist do not provide the level of service for quality improvement reported here. Our approach also differs from the current approach promoted through the Institute for Healthcare Improvement through its "quality-improvement collaborative," which uses multiday learning sessions for health care teams that take place over several months. The teams then apply the principles (i.e., the Plan-Do-Study-Act framework and the Chronic Care Model) acquired during the learning sessions to target interventions for improved diabetes care (25). A recent evaluation of community health centers in Illinois suggested that preventive care for patients with diabetes improved after participation (26). However, rural primary care practices cannot always absorb both the travel costs and time away from clinical care to participate in formally organized sessions. In addition, relatively little on-site support for quality improvement is provided through the collaborative model. The strategies used by the rural practices and state diabetes programs are similar to those described in a comprehensive summary of diabetes quality-improvement techniques, but few of the studies reviewed in the meta-analysis were conducted in rural settings (27).

Although our approach to quality improvement for diabetes care may be unique, other state health departments have built successful relationships with key health systems to improve diabetes care. In Minnesota, the state diabetes program worked with managed care organizations and reported successful efforts not only to improve care but also to develop productive relationships between public health organizations and managed-care partners (28). Such secondary benefits have also been evident in our collaboration with primary care practices in Montana and Wyoming. By working on-site with primary care practices, we were able to identify problems in interpreting laboratory values — such as results of microalbuminuria tests — and address issues in laboratory testing that could impede improvements in diabetes care (29).

### Limitations

There are several limitations to this evaluation. First, time-series analyses were used to evaluate the impact of the interventions on preventive care and HbA1c values, and no comparison group was used. Therefore, it is possible that the improvements we documented were attributable to secular trends. Recent data from the National Health and Nutrition Examination Survey, however, showed that the percentage of adult patients with diabetes with HbA1c levels less than 7.0% decreased between 1988–1994 and 1999–2000 (30). Thus, the improved blood glucose control in our population is probably not a result of secular trends. Second, we assessed changes in preventive care and HbA1c values in the cross-sectional population in these primary care practices. The number of patients with diagnosed diabetes receiving care in these practices increased over time, and there may be variation in the delivery of care for the cohort of patients receiving care compared with the entire patient population over time. However, we found little variation in diabetes care or outcomes between patient cohorts and cross-sectional patient populations assessed previously in similar settings (23). In addition, the reliability of collection and entry of clinical data at primary care practices could have varied over time. Third, the results presented in this report are limited to a few measures that did not include blood pressure or LDL-C levels. Clinical recommendations for blood pressure changed from less than 130/85 mm Hg to less than 130/80 mm Hg during the years when practices were implementing the registries and quality-improvement projects, and LDL-C targets changed from less than 130 mg/dL to less than 100 mg/dL. The aggregate reports provided by participating practices were updated to reflect the changes; therefore, we do not have consistent statewide data for the measures over the time period.

### Solutions, not barriers

Our findings demonstrate that this unique field-based approach to support quality diabetes care in rural states can be successful and can also be sustained for years. This is one of the few reports describing solutions rather than barriers for this important part of the health care system in the United States. Most importantly, this report also provides an example of how public health and primary care can work collaboratively to improve care for people with diabetes.

## Figures and Tables

**Table 1:** Characteristics of Primary Care Practices Participating in a Diabetes Quality-Improvement Program at Follow-up, Montana and Wyoming^a^

**Characteristic**	**Montana**		**Wyoming**	

	**All Practices (n = 24)**	**Practices Participating for ≥6 Months (n = 21)**	**All Practices (n = 16)**	**Practices Participating for ≥6 Months (n = 16)**
Median no. of patients with diagnosed diabetes (range)	117 (21–1646)	123 (21–1646)	136 (36–449)	136 (36–449)

**Type of practice**	**No. (%)**	**No. (%)**	**No. (%)**	**No. (%)**

Multispecialty clinic	5 (21)	5 (24)	0 (0)	0 (0)
Group or individual primary care clinic	11 (46)	8 (38)	15 (94)	15 (94)
Community health center	5 (21)	5 (24)	1 (6)	1 (6)
Urban Indian health center	3 (13)	3 (14)	0 (0)	0 (0)

a Baseline data were collected in 1997 in Montana and in 1998 in Wyoming; follow-up occurred on July 31, 2004.

**Table 2:** Patients With Diagnosed Diabetes Receiving Preventive Care Services and Meeting Selected Clinical Outcomes at Baseline and Follow-up, Montana and Wyoming^a^

**Montana, 1997–2004**						

**Preventive Care Service**	**Median % (Range) Among Practices**			**Overall**		

	**Baseline **	**Follow-up **	***P***	**Baseline % (n/N)**	**Follow-up % (n/N)**	***P* (χ^2^_1_)**
HbA1c test in the past year	76 (40–90)	78 (39–93)	.90	74 (2686/3642)	77 (4532/5859)	.001 (17.6)
Last HbA1c < 8.0%	64 (50–100)	73 (25–91)	.01	66 (1786/2686)	75 (3388/4532)	.001 (56.7)
LDL-C test in the past year	51 (13–86)	52 (25–91)	.04	49 (1775/3625)	59 (3451/5810)	.001 (98.3)
Foot examination in the past year	42 (17–95)	53 (4–80)	.57	45 (1650/3642)	51 (2992/5859)	.001 (29.8)
Dilated retinal examination in the past year	11 (0–37)	25 (0–44)	.14	14 (514/3642)	27 (1592/5859)	.001 (222.0)
Pneumococcal vaccination ever received	25 (0–78)	58 (5–78)	.001	26 (963/3642)	52 (3073/5859)	.001 (621.7)

**Wyoming, 1998–2004**						
**Preventive Care Service**	**Median % (Range) Among Practices**			**Overall**		

	**Baseline **	**Follow-up **	***P***	**Baseline % (n/N)**	**Follow-up % (n/N)**	***P* (χ^2^_1_)**

HbA1c test in the past year	77 (58–100)	78 (58–99)	.71	79 (1239/1566)	79 (1783/2267)	.90 (56.7)
Last HbA1c < 8.0%	78 (60–90)	81 (58–91)	.09	77 (951/1239)	81 (1376/1783)	.92 (0.07)
LDL-C test in the past year	60 (41–83)	63 (33–74)	.91	59 (928/1566)	59 (1334/2267)	.90 (0.07)
Foot examination in the past year	61 (32–100)	52 (3–100)	.23	68 (1066/1566)	58 (1319/2267)	.003 (38.5)
Dilated retinal examination in the past year	13 (0–72)	16 (0–47)	.95	22 (351/1566)	22 (507/2267)	.98 (0.00)
Pneumococcal vaccination ever received	49 (0–90)	56 (11–90)	.002	45 (707/1566)	54 (1230/2267)	.001 (38.8)

a Includes primary care practices participating in the state diabetes quality-improvement program for 6 or more months (n = 37).
